# efam: an *e*xpanded, metaproteome-supported HMM profile database of viral protein *fam*ilies

**DOI:** 10.1093/bioinformatics/btab451

**Published:** 2021-06-16

**Authors:** Ahmed A Zayed, Dominik Lücking, Mohamed Mohssen, Dylan Cronin, Ben Bolduc, Ann C Gregory, Katherine R Hargreaves, Paul D Piehowski, Richard A White III, Eric L Huang, Joshua N Adkins, Simon Roux, Cristina Moraru, Matthew B Sullivan

**Affiliations:** Department of Microbiology, The Ohio State University, Columbus, OH 43210, USA; Center of Microbiome Science, The Ohio State University, Columbus, OH 43210, USA; Max-Planck-Institut fuer Marine Mikrobiologie, Bremen 28359, Germany; Department of Microbiology, The Ohio State University, Columbus, OH 43210, USA; Center of Microbiome Science, The Ohio State University, Columbus, OH 43210, USA; The Interdisciplinary Biophysics Graduate Program, The Ohio State University, Columbus, OH 43210, USA; Department of Microbiology, The Ohio State University, Columbus, OH 43210, USA; Department of Microbiology, The Ohio State University, Columbus, OH 43210, USA; Department of Microbiology and Immunology, Rega Institute, KU Leuven, Leuven 3000, Belgium; VIB-KU Leuven Center for Microbiology, Leuven, Belgium; Department of Microbiology, The Ohio State University, Columbus, OH 43210, USA; Department of Life Sciences, Manchester Metropolitan University, Manchester M1 5GD, UK; Earth and Biological Sciences Directorate, PNNL, Richland, WA 99354, USA; Department of Bioinformatics and Genomics, The University of North Carolina at Charlotte 9201 University City Boulevard, Charlotte, NC 28223, USA; Department of Bioinformatics and Genomics, The University of North Carolina at Charlotte 150 Research Campus Drive, Kannapolis, NC 28081, USA; Australian Centre for Astrobiology, University of New South Wales, Sydney, NSW 2052, Australia; RAW Molecular Systems (RAW), INC, Concord, NC 28025, USA; Earth and Biological Sciences Directorate, PNNL, Richland, WA 99354, USA; Earth and Biological Sciences Directorate, PNNL, Richland, WA 99354, USA; DOE Joint Genome Institute, Lawrence Berkeley National Laboratory, Berkeley, CA 94720, USA; The Institute for Chemistry and Biology of the Marine Environment (ICBM), University of Oldenburg, Oldenburg 26111, Germany; Department of Microbiology, The Ohio State University, Columbus, OH 43210, USA; Center of Microbiome Science, The Ohio State University, Columbus, OH 43210, USA; The Interdisciplinary Biophysics Graduate Program, The Ohio State University, Columbus, OH 43210, USA; Department of Civil, Environmental and Geodetic Engineering, The Ohio State University, Columbus, OH 43210, USA

## Abstract

**Motivation:**

Viruses infect, reprogram and kill microbes, leading to profound ecosystem consequences, from elemental cycling in oceans and soils to microbiome-modulated diseases in plants and animals. Although metagenomic datasets are increasingly available, identifying viruses in them is challenging due to poor representation and annotation of viral sequences in databases.

**Results:**

Here, we establish efam, an *e*xpanded collection of Hidden Markov Model (HMM) profiles that represent viral protein *fam*ilies conservatively identified from the Global Ocean Virome 2.0 dataset. This resulted in 240 311 HMM profiles, each with at least 2 protein sequences, making efam >7-fold larger than the next largest, pan-ecosystem viral HMM profile database. Adjusting the criteria for viral contig confidence from ‘conservative’ to ‘e*X*tremely *C*onservative’ resulted in 37 841 HMM profiles in our efam-XC database. To assess the value of this resource, we integrated efam-XC into VirSorter viral discovery software to discover viruses from less-studied, ecologically distinct oxygen minimum zone (OMZ) marine habitats. This expanded database led to an increase in viruses recovered from every tested OMZ virome by ∼24% on average (up to ∼42%) and especially improved the recovery of often-missed shorter contigs (<5 kb). Additionally, to help elucidate lesser-known viral protein functions, we annotated the profiles using multiple databases from the DRAM pipeline and virion-associated metaproteomic data, which doubled the number of annotations obtainable by standard, single-database annotation approaches. Together, these marine resources (efam and efam-XC) are provided as searchable, compressed HMM databases that will be updated bi-annually to help maximize viral sequence discovery and study from any ecosystem.

**Availability and implementation:**

The resources are available on the iVirus platform at (doi.org/10.25739/9vze-4143).

**Supplementary information:**

Supplementary data are available at *Bioinformatics* online.

## 1 Introduction

Marine viruses infecting microbes are the most abundant biological entities on the planet (Hendrix *et al.*, 1999). With abundances of up to 10^11^ per liter of seawater and approaching 2 × 10^5^ viral populations or ‘species’ cataloged across the global ocean (Gregory *et al.*, 2019), viruses outnumber their hosts in abundance and potentially in diversity (Ignacio-Espinoza, 2013). Viruses affect their host’s metabolism, physiology, evolution and mortality and consequentially alter global biogeochemical cycles. They are a major force driving nutrient cycling (Suttle, 2007) and are thought to impact carbon fluxes in the oceans, making them critical players in global climate regulation (Guidi* et al*., 2016). Despite their impact, much of the viral sequence space remains to be discovered. The primary methodology for discovery is high throughput metagenomic sequencing. This is because the vast majority of viruses are not in culture, with only 15% of the known prokaryotic phyla having cultured representatives at all (Roux *et al.*, 2015b) and viral isolation is dependent on the ability to grow their host. Additionally, the discovery and functional annotation of viral contigs in cellular metagenomes—or even in viral metagenomes, commonly characterized by a high non-viral background (Roux *et al*, 2013)—utilizes tools that ultimately rely upon known viral sequences.

Hence, several Hidden-Markov-Model (HMM) profile databases have been developed and utilized for annotating viral genes for functional characterization in metagenomes. HMM databases increase the sensitivity of homology identification, which is suitable for what is posited to be a broad and expansive viral sequence space (Soding, 2005). Existing databases were developed from (i) reference sequences of viral isolates and prophages (e.g. RefSeq), such as POGs/pVOGs (Kristensen, 2013;  Grazziotin, 2017), uPOGs (Zheng *et al.*, 2019), vFams (Skewes-Cox *et al.*, 2014) and VOGDB (http://vogdb.org/), (ii) reference sequences (e.g. UniProt Knowledgebase) of cellular or viral origin, such as Pfam (El-Gebali *et al.*, 2019) and TIGRFAMs (Haft *et al.*, 2013) or (iii) viral reference sequences and curated viral contigs from metagenomes, such as VPF (Paez-Espino *et al.*, 2016). However, all these databases underrepresent marine viruses for the reasons outlined above.

Here, we developed efam and efam-XC, as conservative and extremely conservative databases, respectively, representing expanded and annotated HMM profiles of viral protein families recovered from the Global Ocean Virome 2.0 (GOV 2.0) dataset (Gregory *et al.*, 2019), to aid viral discovery and functional annotations, particularly from marine ecosystems. GOV2 is unprecedented with regard to both sequencing depth and geographic scope including sequences from ∼200K viral populations (approximately viral ‘species’; Gregory *et al.*, 2016, 2019; Roux *et al.*, 2019a,b) from different ocean layers and all major ocean basins, including geopolitically challenging circumpolar sampling from the Arctic Ocean. Profile annotations were performed using both sequence similarity-based, confidence-scored bioinformatic methods and dedicated metaproteomic experiments designed to identify proteins associated with highly purified viral particles, while performance tests revealed significantly improved viral detection across multiple viromes.

## 2 Materials and methods—construction of efam and efam-XC

### 2.1 Selection of highest-confidence viral contigs from the GOV 2.0 dataset

The GOV2.0 dataset (Gregory *et al.*, 2019), containing 848 507 viral contigs, was used as a source of novel marine viral genomes. A length threshold of ≥5 kb was used for linear contigs, and of ≥1.5 kb for circular ones. We re-analyzed all GOV 2.0 contigs using three different viral prediction tools: VirSorter (Roux *et al.*, 2015a), DeepVirFinder (Ren *et al.*, 2020) and MARVEL (Amgarten *et al.*, 2018). VirSorter was run in the ‘virome decontamination’ mode, choosing the virome database as the reference. DeepVirFinder and MARVEL were run using the default parameters. The strictest cut-offs for viral contig detection were used to select sets of viral contigs with the highest confidence scores according to each tool’s classification model, as follows: (i) category 1 for VirSorter; (ii) score > 0.9 and *P*-value < 0.05 for DeepVirFinder and (iii) score > 90% for MARVEL. Additionally, only contigs predicted by all three tools (efam-XC) or by at least two of them (efam) were selected. Further, each dataset was cleaned from non-viral regions by using CheckV’s contamination module (Nayfach *et al.*, 2020).

### 2.2 Protein prediction, decontamination and clustering

For each contig, the open-reading-frames (ORFs) and their corresponding proteins were predicted using Prodigal in metagenomic mode (Hyatt *et al.*, 2010). The resulting proteins were then searched against a RefSeq-database containing all bacterial and archaeal proteins (access date: January 12, 2019) using Diamond BLASTP (Buchfink *et al.*, 2015). Sequences that showed high local identity (percent identity > 95% with no minimum coverage requirement and considering all the hits rather than just the top hit) to bacterial and archaeal proteins were excluded. Exceptions were the proteins with one of the following keywords in its annotation: ‘tail’, ‘capsid’, ‘portal’, ‘virus’, ‘virion’, ‘viral’, ‘phage’, ‘bacteriophage’ or ‘terminase’, detected by using the string search (str_detect) function of package ‘stringr’ (Wickham, 2017) in R (R Core Team, 2020). Subsequently, the remaining proteins were dereplicated (at 100% identity) using USEARCH (Edgar, 2010) with the –fastx_uniques flag.

The selected protein sequences were clustered by (i) running an all-against-all Diamond BLASTP search using the default parameters (except for using the –more-sensitive flag), (ii) filtering out matches with an evalue >10e-5, coverage <70% of the length of either sequence [to reduce regions with gaps in the multiple sequence alignment step below (*sensu* the vFams pipeline; Skewes-Cox *et al*., 2014)], and with total length <50 amino acids for either sequence (to minimize including mis-called proteins in the Prodigal step above as well as incomplete protein sequences), (iii) performing a negative log10 transformation on the e-values (to make the values directly proportional to sequence similarity) and then, removing any non-positive transformed e-values and applying a ceiling of 200 on the remaining transformed e-values (i.e. treating all cases with an e-value < e-200 the same) and (iv) clustering the matching protein pairs (nodes) based on the final transformed e-values (edges) using the graph-based clustering method in ClusterONE (Nepusz *et al.*, 2012). The default parameters for ClusterONE were used, except for the minimum sequence number per cluster (-s 2). In order to examine the impact of the clustering software on the final cluster sets, we also clustered the proteins using MCL (Enright *et al.*, 2002), using the transformed e-values as edge weight and the default parameters of MCL.

### 2.3 Creation of Hidden-Markov-Model profiles and bioinformatic annotations

Sequences within each cluster were aligned using MUSCLE (Edgar, 2004), with a maximum number of iterations of 4 (–maxiters = 4), to balance accuracy and speed. Then, HMM profiles were built for each of the multiple-sequence-alignments using hmmbuild, which is part of the HMMER3 package (Eddy, 1998), with the default parameters. All HMM profiles were compressed into a HMMER3 searchable database, using hmmpress (from HMMER3 package). This allows for quick searches of the database with hmmscan or similar programs.

The proteins in each cluster were annotated using the ‘annotate_genes’ module of DRAM (Shaffer *et al.*, 2020) against KEGG (Kanehisa *et al.*, 2016), UniRef90 (Suzek *et al.*, 2015), Pfam (El-Gebali *et al.*, 2019) and VOGDB (http://vogdb.org/). Information about each cluster, including cluster number, the number of proteins within the cluster and the confidence/rank of the annotations are included in the companion tables of the searchable HMM databases.

### 2.4 Annotations of virion-associated proteins using metaproteomic data

A total of 48 viral metaproteomes from the *Tara* Oceans expeditions were used to further annotate viral structural proteins in efam and efam-XC. Twelve metaproteomes were previously generated from 4 samples (Brum* et al*., 2016) and 36 were generated in this study, from 33 samples, and analyzed using the mass spectrometry platform described in Huang *et al.* (2016). The sampling dates, locations and depths for all the viral metaproteomes are provided in (Supplementary Table S1). The metaproteome spectral files were queried for the presence of GOV 2.0 proteins, as follows. Spectral input files were first converted from .RAW to .mzML using msConvert of ProteoWizard 3.0.10200 (Chambers *et al.*, 2012), using the default parameters. Spectral files were then searched using MSGFPlus v2017.01.13 (Kim and Pevzner, 2014) against all protein sequences from the GOV2.0. MSGFPlus searches were performed with the following parameters: ±20 ppm parent mass tolerance; isotope error range (-ti ‘-1,2’); fully tryptic enzyme settings (-e 1 -ntt 2); 6 and 50 as the minimum and maximum peptide lengths to consider, respectively; reporting only the Peptide-Spectral Matches (PSMs) with the highest MSGF score (-n 1); conducting a parallel search against a decoy protein database (-tda 1) for calculating the false discovery rate (FDR). After conducting the searches, the FDR was calculated as described previously (Woodcroft *et al.*, 2018) and an FDR cutoff of 1% was applied for each independent search.

All detected peptides (FDR ≤ 1%) were mapped to the dereplicated set of proteins that comprise efam and efam-XC using the PeptideToProteinMapper console app v1.3.6794 (omics.pnl.gov/software/protein-coverage-summarizer) with flags ‘\G\H\A’. The set of proteins and clusters that recruited each peptide were collapsed and condensed in a single table, adding the statistics of these mapped proteins and clusters and the DRAM annotations to each cluster along with any additional information on these annotations, such as whether it was previously annotated and/or had a structural annotation. A cluster was considered previously annotated if its annotations from KEGG, UniRef90, Pfam or VOGDB did not include any the following keywords: ‘uncharacterized’, ‘hypothetical’, ‘no annotation’ or ‘duf’ (short for domain of unknown function). Among clusters with previous annotations, a cluster was considered to have a previous structural annotation if it included any of the following keywords: ‘coat’, ‘capsid’, ‘virion’, ‘head’, ‘neck’, ‘mu’, ‘fiber’, ‘tail’, ‘sheath’, ‘structur*’ (for structure or structural), ‘spike’, ‘baseplate’, ‘gp23’ (major capsid protein), ‘gp9’ (baseplate protein). All searches against these keywords were case insensitive.

### 2.5 Benchmarking viral discovery in metagenomes with efam-XC

In order to assess the potential contribution of efam-XC towards the detection of unknown viral sequences, VirSorter was run on two sets of marine metagenomes—both of which were not part of building efam or efam-XC, and both are ecologically distinct from open ocean viromes (Vik *et al.*, 2021)—as well as two sets of non-marine metagenomes from the well-studied human gut (Gregory *et al.*, 2020; Shkoporov *et al.*, 2019) and less-studied permafrost soils (Roux *et al.*, 2019a). The first marine dataset comprised of 28 viromes, collected from the Eastern Tropical South Pacific oxygen minimum zone (ESTP-OMZ; Vik *et al.*, 2021). The second marine dataset represented a deeply re-sequenced virome from waters of the LineP transect (Hurwitz and Sullivan, 2013). The sampling dates, locations and depths for all the samples in these two marine datasets and the details about the samples from the gut and permafrost soil datasets are provided in (Supplementary Table S2). VirSorter was run twice on these metagenomes, one time using the built-in ‘Virome’ database and one time using a combined ‘Viromes + efam-XC’ database, while leaving all other parameters the same. Subsequently, the number of identified viral contigs (considering all of the VirSorter categories) was compared for each run. Finally, the newly identified contigs (due to the addition of efam-XC) were assessed for their viralness using DeepVirFinder, MARVEL and CheckV.

## 3 Results and discussion

### 3.1 efam and efam-XC vastly extend the viral protein sequence space organized into databases

**Fig. 1.**
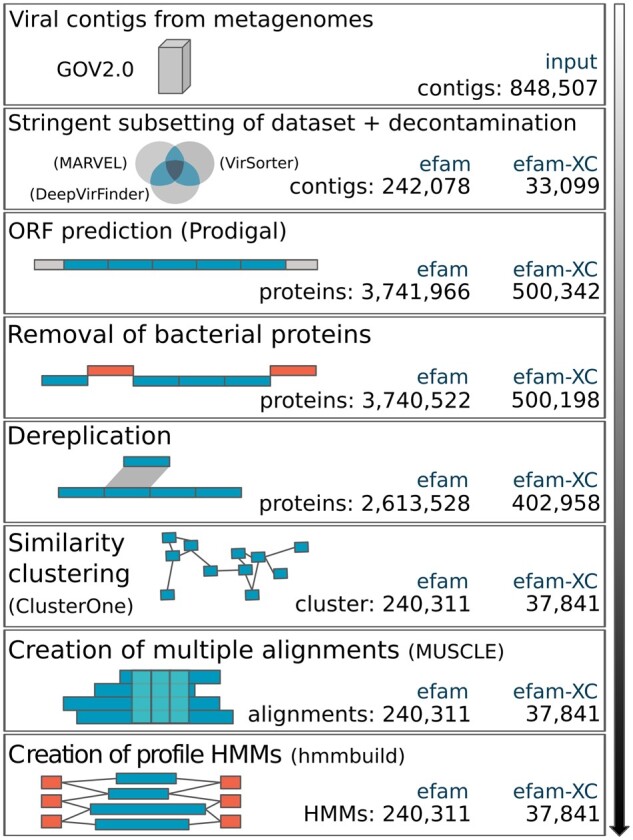
Computational workflow used to construct efam and efam-XC. This pipeline illustrates the major steps (rectangles) followed to generate efam and efam-XC. Whenever applicable, software used in each step are shown in parentheses. Viral sequences from GOV2.0 were re-analyzed by three different viral prediction tools and extremely conservative subsets of these predictions were used downstream after decontamination (i.e. removal of prokaryotic genes from any potential prophage contig) by CheckV. Open reading frames (ORFs) on each viral contig were predicted and the protein sequences that were 95% locally similar to bacterial or archaeal proteins were removed. The remaining proteins were then dereplicated and clustered, and the sequences within each cluster were multiple aligned. Finally, HMM profiles were built out of each alignment and the profiles were pressed into a searchable HMM database. Statistics for each step in the generation of the efam and efam-XC are shown in the bottom right corner of each box

Given our interest in maximizing marine viral protein sequence space organization, we focused on generating efam and efam-XC with data from the GOV 2.0 dataset (Gregory *et al.*, 2019). GOV 2.0 includes 145 deeply sequenced viromes derived from water samples distributed throughout the world's oceans, from pole to pole and collected from the epi-, meso- and bathy-pelagic oceanic layers (0–150 m, 150–1000 m, >2000 m, respectively). Therefore, it also includes less sampled, but highly relevant marine environments, such as the Arctic Ocean and the deep ocean. In total, GOV2.0 contains 848 507 viral contigs with lengths ranging from 1.5 to 500 kb, and an average of **∼**44 kb (Gregory *et al.*, 2019). On these data we applied 3 layers of stringent selection to maximize the confidently identified viral content of efam and efam-XC (Fig. 1).

**Fig. 2.**
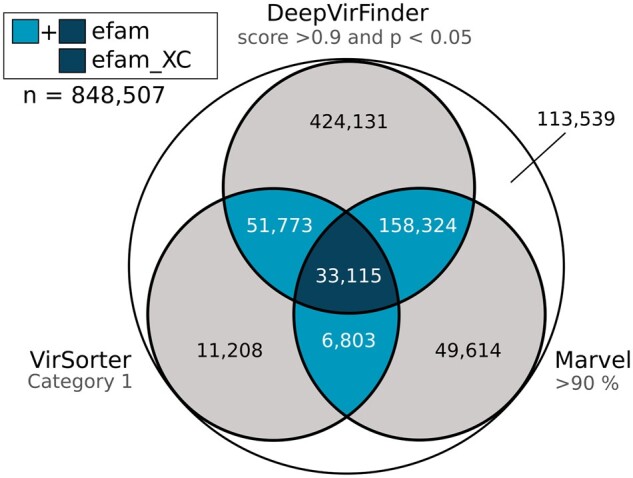
Stringency levels used for selecting the viral contigs contributing to efam and efam-XC. The Venn-diagram shows the extent of agreement between VirSorter, DeepVirFinder and MARVEL at the highest stringency levels of each program. The intersection of the highest stringency of at least two programs was used to construct efam, while the intersection of the highest stringency of all three programs was used to construct efam-XC

First, the GOV 2.0 dataset was subjected to three complementary virus prediction tools, applying the most stringent cutoffs from each tool (see Section 2). To build efam-XC, we used only those contigs predicted by all the three tools (Fig. 2; see Section 2 for details). To build efam, we used the contigs predicted by at least two of the three tools to override the limitations of each individual tool. For example, DeepVirFinder and MARVEL can capture shorter contigs (Amgarten *et al.*, 2018; Pratama *et al.*, 2021; Ren* et al*., 2020), a limitation of VirSorter. VirSorter, on the other hand, is better able to capture viral contigs that have similar K-mer signatures to their hosts, a limitation of DeepVirFinder, with lower false positive rates than the other tools when mobile elements are present in datasets (Pratama *et al.*, 2021; Ren *et al.*, 2020; Roux *et al.*, 2015a). Out of all GOV 2.0 contigs, only 29% (*n* = 250 021) met the stringent criteria required for efam, and the number decreased to 4% (*n* = 33 115) upon using the extremely stringent criteria for efam-XC (Fig. 2). Most of the contigs that were exclusively recognized by only one program at the highest confidence level were captured at a lower confidence level by at least one of the other two programs (Fig. 2). This leaves room for the inclusion of a larger number of viral contigs into future versions of efam and efam-XC, as new algorithms arise (e.g. VIBRANT, VirSorter2) that may improve viral detection and confidence assessments (Guo *et al.*, 2021; Kieft *et al.*, 2020). Notably, the stringent selection of contigs in this first layer did not strongly bias the viral taxonomic groups included in the efam and efam-XC databases towards Caudovirales, a viral order that is over-represented in reference databases. This is because cross-referencing efam and efam-XC viral contigs with the taxonomic classification from GOV2.0 (Gregory *et al.*, 2019) showed that only 18.4% and 27% of the input viral populations used in our databases, respectively, come from Caudovirales (compared to the 9.8% Caudovirales viral populations in GOV2.0). Hence, even though there is a slight bias towards Caudovirales (especially for the efam-XC database), our resources go beyond this order and should be of greater benefit to the future users.

The second and third layers of selection leveraged detection of contamination due to similarity to ‘prokaryotic’ databases. Specifically, the selected contigs were subjected to decontamination using CheckV (Nayfach *et al.*, 2020), which identifies contaminants based on detection of ‘prokaryote only’ proteins in HMM profile searches, removing host genes from prophage contigs (Nayfach *et al.*, 2020) and potentially contigs with long stretches of Auxiliary metabolic genes (Shaffer *et al.*, 2020). This removed only **∼**3.2% (*n* = 7 943) and **∼**0.05% (*n* = 16) potential prophage contigs from efam and efam-XC, respectively, leaving 242 078 (efam) and 33 099 (efam-XC) contigs remaining (Fig. 1). Finally, a third layer of selection was applied at the protein level, to remove those that have local similarity to bacterial or archaeal analogs (see Section 2) to avoid recruiting those prokaryotic proteins by the final database due to short sequence (e.g domain) matches. In total, 3 741 966 and 500 342 proteins were predicted from the remaining efam and efam-XC contigs above, respectively. A local alignment search against bacterial and archaeal proteins followed by the removal of strong matches resulted in the removal of only **∼**0.04% (*n* = 1444) and **∼**0.03% (*n* = 153) proteins from efam and efam-XC, respectively. This was followed by a dereplication step (see Section 2) that resulted in the removal of **∼**30% (*n* = 1 126 994) and **∼**19.5% (*n* = 97 231) proteins, leaving 2 613 528 (efam) and 402 958 (efam-XC) unique proteins remaining. Clustering these remaining proteins resulted in 240 311 (efam) and 37 841 (efam-XC) non-singletons protein clusters (number of non-included singletons was 300 021 and 56 148, respectively; Supplementary Table S3). The entire overview of this workflow is outlined in (Fig. 1).

**Fig. 3.**
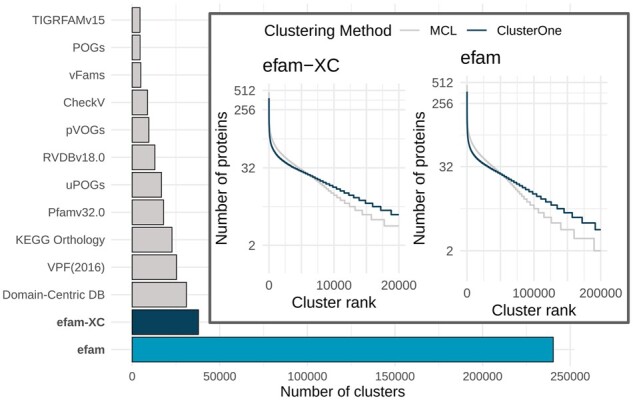
Comparison of viral HMM database sizes and clustering algorithms. Number of clusters (HMM profiles) in efam, efam-XC and currently available public databases. SFams, another HMM profile database (Sharpton *et al.*, 2012), was excluded from our comparisons because it did not include any viral genomes in its construction. (Inset) Clustering structure produced by ClusterOne and MCL for efam (right) and efam-XC (left). The number of clusters on the x-axes were capped at 200 000 (efam) and 20 000 (efam-XC) for visibility. ClusterOne generally produced longer tails (more clusters) and larger clusters except for the highly ranked clusters. Since ClusterOne was instructed to apply a ‘hair-trimming’ step after the clustering to remove dangling nodes and since the highly ranked clusters have more representative sequences that are used to build the HMM profiles, we felt comfortable proceeding with well-trimmed slightly smaller high-rank clusters. The number of protein sequences used for clustering in each database is listed in (Supplementary Table S4)

In their nascent iterations, efam and efam-XC represent a big contribution to the viral protein sequence space organized into HMM databases. In addition to better capturing previously underrepresented marine viral sequences (see below), the sheer scale of the databases was some of the largest in comparison to previously established databases (Fig. 3 and Supplementary Table S4). Specifically, efam-XC is currently the largest viral HMM profile database available; it exceeds VPF **(**from IMG/VR v.2.0); built from viral contigs found in habitats throughout the planet (Paez-Espino *et al.*, 2016), uPOGs—the recently updated version of pVOGs (Zheng *et al.*, 2019), vFams (Skewes-Cox *et al.*, 2014) and all other currently known databases as listed in (Fig. 3). Notably, efam, which still conservatively captures viral sequence space, is more than 6-fold and 7-fold larger than efam-XC and the next largest publicly available database (VPF), respectively. To assess whether our new databases were larger merely due to newer clustering algorithms or to the data underlying them, we re-clustered our underlying efam and efam-XC datasets using MCL, the clustering software used by VPF and vFams. This revealed the same patterns of database size distribution after re-clustering (Fig. 3). Thus, while ClusterOne produced slightly more clusters than MCL for both efam and efam-XC and, in both cases, most of the clusters produced by ClusterOne contained more proteins (Fig. 3), it was not the dominant factor driving database size in this study. Instead, the workflow that we introduced before the protein clustering step (Fig. 1) to maximize the confidence in the ‘viralness’ of proteins from our new and highly diverse environmental viromes (Gregory *et al.*, 2019), leveraged by modern viral identification tools that were largely not available to other databases at the time of their inception (Fig. 2), allowed for establishing efam and efam-XC as much expanded resources over existing databases. This workflow, along with the workflow we introduced for annotating the protein clusters both *de novo* and using multiple databases (see below) differentiates efam and efam-XC from previously published efforts. The methods described here can be directly applied to other viromes and other ecosystems, perhaps with the exception of viral-targeted metaproteomes (to annotate the protein clusters *de novo*) that may be difficult to generate from more complex ecosystems such as soils.

### 3.2 efam and efam-XC boost viral discovery in metagenomes

**Fig. 4.**
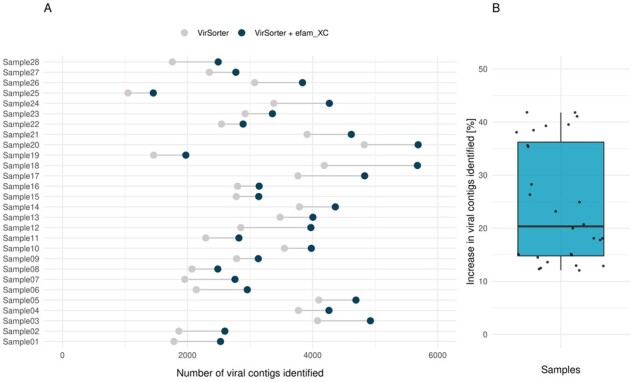
efam-XC enables viral discovery in metagenomes. The paired dot plot (**A**) shows that the number of recovered viral contigs from every single ETSP-OMZ virome increased upon integrating efam-XC in VirSorter. As a result, the median and average number of viral contigs recovered per sample (**B**) increased for the new implementation of VirSorter, with the average increasing from 2904 to 3558 viral contigs per sample (22.5% increase)

**Fig. 5.**
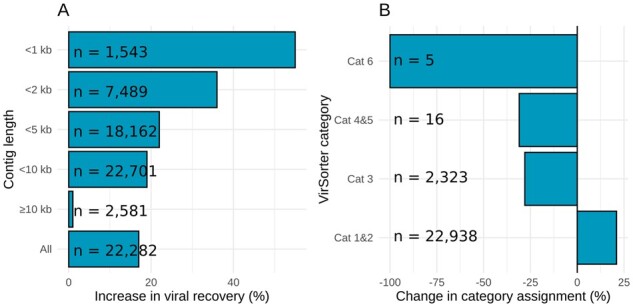
efam-XC enhances the recovery of short viral contigs and increases confidence level in identified contigs. (**A**) Percent increase in the number of viral contigs recovered by VirSorter from the deeply sequenced LineP sample at different contig sizes upon integrating efam-XC into VirSorter. (**B**) Percent decrease in the number of low-confidence viral contigs (Cat 3 and Cat 6 of VirSorter) and the prophage category (Cat 5) upon integrating efam-XC into VirSorter. The viral contigs that were removed from these categories were added to the high-confidence categories (Cat 1 and Cat 2), except for 2 contigs from Cat 6 which were moved to Cat 3. The numbers next to each bin are from the VirSorter run before integrating efam-XC

The identification of viral contigs within metagenomes is largely reliant on sequence similarity searches against a database of known viral sequences. Therefore, we hypothesized that augmenting the reference databases of viral identification tools with efam or efam-XC would improve detection sensitivity in new metagenomes. To assess this, we augmented the ‘viromes’ database of VirSorter with efam-XC, and found that, indeed, it consistently increased the number of identified viral contigs across all virome samples collected from new marine virome datasets from the Eastern Tropical South Pacific oxygen minimum zone (ESTP-OMZ; Fig. 4) and the LineP transect (Fig. 5). These viromes were selected to be performance test samples because (i) they were not part of the contig pool used to establish efam and efam-XC, and (ii) their viruses were only distantly related to open ocean viruses (Vik *et al.*, 2021). The percent increase in viral contig recovery ranged from to 12.1% to 41.8% (with an average of **∼**24%) more contigs, depending on the sample. These new contigs are unlikely to be microbial in origin because 88% (17 307 out of 19 654) of them were also called viral by DeepVirFinder (score > 0.7 and *P*-value < 0.05) or MARVEL (>70% probability score), and 98.2% (19 306 out of 19 654) of them had 0% contamination (with only 97 contigs having ≥ 50% contamination) using CheckV. For the LineP sample, a deeply sequenced sample collected off the coast of British Columbia, the number of identified viral contigs increased by 16.8%, while for non-marine samples [from the more-studied human gut (*n* = 3) and less-studied permafrost soils (*n* = 3)], the increase was on average 2.7% and 7.1%, respectively. Even though the current version of the database proved useful to other ecosystems, future versions will integrate viruses from a variety of ecosystems, including a large number of novel viruses currently under investigation by our group, to maximize discovery.

We next wanted to understand the nature of the new viruses being identified, with particular focus on smaller contigs from the deeply sequenced LineP virome. Due to the deeper sequencing, a high number of smaller contigs were assembled for this sample, which we examined further. First, integrating efam-XC into VirSorter allowed for the detection of higher proportions of the shorter contigs (Fig. 5), which has been demonstrated to be especially challenging to the current implementation of VirSorter (Ren *et al.*, 2020). However, short contigs, which constitute the majority of metagenomic assemblies, facilitate viral gene ecology analyses and recruit considerably more reads to viral sequences in viromes due to their dominance in the assembly (Mende *et al.*, 2012). In the deeply sequenced LineP sample, we found that the percent increase in viral recovery for contigs increased inversely with contig length (Fig. 5A). This directly highlights the gaps in previous viral reference protein sequence space that were traditionally compensated by VirSorter’s ability to detect other well-known viral proteins on the longer contigs. Finally, in the LineP sample, we observed that inclusion of efam-XC data into VirSorter overall improved the quality of viral prediction, because many low-confidence virus contigs (60% of ‘Cat 6’ viral contigs and 19.4% of ‘Cat 3’ viral contigs) were assigned to the higher confidence categories ‘Cat 1’ or ‘Cat 2’ when using efam-XC, with the remaining of Cat 6 (40%) reassigned to ‘Cat 3’ (Fig. 5B;  Supplementary Table S5). Thus, the information efam-XC added to such viral contigs increased the confidence calls by VirSorter’s probabilistic model. Similarly, 56.3% of ‘Cat 5’ viral contigs—a high confidence assignment by VirSorter for potential prophages—were assigned to Cat 2, indicating that efam-XC leveraged VirSorter’s ability to better resolve gene assignments at the edges of the viral contigs and move them away from a possible prophage category (Fig. 5B;  Supplementary Table S5). Notably, a small percentage (2.9%) of the contigs detected by VirSorter were not detected or downgraded to ‘Cat 3’ or ‘Cat 5’ after adding efam-XC (Supplementary Table S5). This can be attributed to the higher sensitivity of the HMM profiles in efam-XC than the default ‘hallmark’ gene profiles in VirSorter, impacting the call of ‘viral’ by VirSorter (Roux *et al.*, 2015a,b). The annotations that we provide here (see below) should help with the updating of the ‘hallmark’ gene list in future versions of VirSorter (e.g. VirSorter2; Guo *et al.*, 2021). To maximize recovery without updating VirSorter’s underlying data, users should use VirSorter, then ‘VirSorter + efam/efam-XC’ and combine the results.

### 3.3 Improved viral profile functional annotation

Beyond identifying viruses, we hoped that the efam and efam-XC databases could offer a step towards a centralized resource with improved functional annotations. This would be critical for multiple facets of virome research including identifying genes that hold the potential to impact host metabolism during the virus-infected or ‘virocell’ stage that can alter biogeochemical cycles and ecosystem outputs of a cell (e.g. Forterre, 2011; Howard-Varona *et al.*, 2020; Roux *et al.*, 2016), as well as identifying viral hallmark genes that increase the confidence in detecting previously unseen viruses in metagenomic sequences (see category 1 viruses in VirSorter; Roux *et al.*, 2015a). Problematically, viral gene annotation has proved to be challenging as the field has a history of observing that most viral genes in metagenomes typically cannot be annotated (Boratto *et al.*, 2020; Gregory *et al.*, 2019; Roux *et al.*, 2016, 2019b). Part of this lack of annotation is due to challenges of scaling annotation, which limits many studies to interrogating against only a single functional annotation database like KEGG or VOGDB.

To provide an improved viral protein resource, we annotated efam and efam-XC via (i**)** DRAM, a rigorous multi-database-supported annotation pipeline designed for both microbial and viral genomes, and (ii**)** identifying previously unknown virion-associated proteins using mass-spectrometry-based metaproteomic measurements made directly on viral particles (Brum *et al.*, 2016). First, DRAM annotated about one-third of the 240 311 (efam) and 37 841 (efam-XC) HMM profiles, with functional annotations (excluding ‘hypothetical proteins’, ‘uncharacterized proteins’ and ‘domains of unknown functions’; see Section 2) provided for 33.5% (*n* = 80 431) and 38.3% (*n* = 14 492), respectively (available at: doi.org/10.25739/9vze-4143). This almost doubled the number of annotations retrievable using a single database such as KEGG (40 707 and 6819 for efam and efam-XC, respectively) or VOGDB (35 664 and 7136 for efam and efam-XC, respectively) at the same parameters and cut-offs used in DRAM. Notably, there was a statistically significant difference between the medians of the cluster sizes for the annotated (median = 6) and unannotated (median = 3) clusters in the efam database (*P-*value ≤ 1e-04), suggesting that larger clusters tend to be more amenable to annotation. For each protein cluster giving rise to an HMM profile, all database annotations of the cluster members were collected and collapsed to different levels of detail; we provide detailed annotations, each with its source database specified, as well as the collective annotation(s) agreed upon by different databases (both available at: doi.org/10.25739/9vze-4143). These annotations (per predicted protein and per protein cluster) are formatted as companion metadata tables to the searchable efam and efam-XC databases that can be queried by the cluster or protein ID. Second, peptides from the viral metaproteomes mapped to **∼**3.7% (*n* = 8847) and 8.6% (*n* = 3262) of the protein clusters of efam and efam-XC, respectively. Therefore, these protein clusters and their member protein sequences were annotated as virion-associated (*sensu* Brum *et al.*, 2016). This effort complemented DRAM annotations (i.e. solved the cases that were not annotated by DRAM) by adding a *de novo* annotation of ‘virion-associated protein’ to 33.7% (*n* = 2984) and 28.1% (*n* = 916) of protein clusters matched by a mass-spec detected peptide from efam and efam-XC, respectively (see Section 2). Notably, for all cases in which DRAM provided annotation for a metaproteome-detected protein cluster (*n* = 5863 and 2346), very few (2.8% and 1.5% of the protein clusters for efam and efam-XC, respectively) were annotated as something other than ‘structural protein’ (see Section 2 and metadata tables at doi.org/10.25739/9vze-4143). This large concordance where DRAM and metaproteomic evidence overlap boosts the confidence of the new annotations inferred from the metaproteomes. We also provide these metaproteome annotations in the companion metadata tables to efam and efam-XC (available at: doi.org/10.25739/9vze-4143).

## 4 Conclusions

Microbiome researchers have helped bring out the myriad and significant roles that microbes play in diverse ecosystems, with many of these advances attributable to better ‘seeing’ microbes as sequencing technologies illuminated the unseen majority often termed ‘microbial dark matter’ (Rinke *et al.*, 2013). A similar revolution is happening for viruses, whereby we are clearly immersed in a ‘third age of phage’ (Mann, 2005) that is being complemented by advances across all viral types with recent surveys in the oceans alone exposing widespread and hidden endogenous viruses (Moniruzzaman *et al.*, 2020), non-tailed viruses (Kauffman *et al.*, 2018), giant viruses (Schulz *et al.*, 2020) and RNA viruses (Wolf *et al.*, 2020). Complementarily, the toolkit to assess viral impacts is expanding rapidly with recent advances including scalable assessment of resistance mechanisms (Mutalik *et al.*, 2020), multi-omics views of how virus-infected cells change their ecosystem outputs (Howard-Varona *et al.*, 2020) and high-throughput detection of virus-host linkages (Bickhart *et al.*, 2019; Deng *et al.*, 2014; Džunková *et al.*, 2019). Here, by organizing and thoroughly annotating the largest ocean virus survey data to date into the efam and efam-XC databases, we hope to have taken one more step forward in these efforts to increasingly expand our window into the wild to better see and understand the roles that viruses play in complex communities. Future implementations of efam and efam-XC will benefit from expanding environmental diversity and adding evolutionary information (*sensu* pVOGs), as well as integration into modern cyberinfrastructures and databases (Bolduc *et al.*, 2017). efam and efam-XC are planned to be updated bi-annually as funding allows and will be kept freely accessible with no restrictions on use.

## Supplementary Material

btab451_Supplementary_Data
